# N6-Methyladenosine Regulator-Mediated Immue Patterns and Tumor Microenvironment Infiltration Characterization in Glioblastoma

**DOI:** 10.3389/fimmu.2022.819080

**Published:** 2022-03-11

**Authors:** Wu Xiong, Cong Li, Bowen Wan, Ziyang Zheng, Yingfei Zhang, Siming Wang, Jin Fan

**Affiliations:** ^1^ Department of Orthopaedics, The First Affiliated Hospital of Nanjing Medical University, Nanjing, China; ^2^ Nanjing Medical University, Nanjing, China; ^3^ Department of Orthopaedics, Subei People’s Hospital of Jiangsu, Clinical Medical College of Yangzhou University, Yangzhou, China

**Keywords:** m6A, glioblastoma, tumor microenvironment, immunotherapy, biomark

## Abstract

**Background:**

Epigenetic modifications, according to emerging evidence, perform a critical role for cellular immune response and tumorigenesis. Nonetheless, the role of N6-methyladenosine modification in shaping of the glioblastoma tumor microenvironment is unknown.

**Methods:**

N6-methyladenosine(m6A) methylation patterns in GBM patients were evaluated *via* multiple omics analysis of 15 m6A regulators and systematically correlated with tumor immune features. For quantification of N6-methyladenosine methylation patterns of individual patients, GM-score was developed and correlated with clinical and immunological characteristics.

**Results:**

Glioblastoma has two different m6A methylation patterns that are strongly associated with TME characteristics, tumor subtype, immunotherapy response, and patient prognosis. High-GM-score is associated with an immune tolerance phenotype dominated by the IDH1 wild molecular subtype and the Mesenchymal tissue subtype, as well as a high infiltration of immune cells and stromal cells and a poor prognosis. Furthermore, despite higher immune checkpoint expression, individuals with a high-GM-score have a poorer response to anti-CTLA4 immunotherapy regimens due to T-cells dysfunctional. Low-GM-score individuals had an immunodeficient phenotype dominated by IDH mutant molecular subtypes and Proneural tissue subtypes, with less immune cell infiltration and a better prognosis. Furthermore, patients with low-GM-scores had higher microsatellite instability (MSI) and t-cell exclusion scores, as well as a better response to anti-CTLA4 immunotherapy regimens.

**Conclusion:**

This study demonstrated that m6A modification patterns play an important role in the shaping of TME complexity and diversity. The GM-score could identify m6A modification patterns in individual patients, resulting in a more personalization and efficacious anti-tumor immunotherapy strategy.

## Introduction

Epigenetic modifications, according to emerging evidence, play a critical role in cellular immune response and tumorigenesis (1). As the most common post-transcriptional modification of RNA, m6A methylation modificationization has recently become a key area of cancer research. The m6A methylation post-transcriptionally influences various biological processes, including RNA processing, splicing, stabilization, translation, and degradation (2, 3). Moreover, m6A methylation is a dynamic, reversible modification process governed by writers, readers and erasers (4, 5). Writers (methyltransferases) such as WTAP, METTL3, RBM15 and ZC3H13, create m6A marks (6). Erasers, mainly FTO, are demethylases (7). Readers, which include YTHDCs, HNRNPC, FMR1 and IGF2BPs, detect and bind m6A modification sites to create equivalent signals (8).

The tumor microenvironment (TME) performs a major effect on cancer progression, according to growing evidence (9). Cancerous cells, mesenchymal cells, infiltrating leukocytes, and secretory factors make up the tumor microenvironment, and it offers a fresh look at tumorigenesis (10). The understanding of the TME landscape grows in tandem with the diversity and complexity of the TME landscape. TME appears to make a significant contribution in the procedure and immunotherapeutic reaction in tumorigenesis, according to a large body of evidence (11). Immunotherapy reactivates the anti-tumor immune response by blocking co-inhibitory receptors (12). Immunotherapy is effective in glioblastoma, but its efficacy has been inconsistent due to a lack of systematic understanding of the glioblastoma TME. As a result, a thorough understanding of TME’s heterogeneity and complexity is critical for identifying new therapeutic targets and evaluating immunotherapeutic response.

In recent years, the role of m6A methylation in TME has received increasing attention (13). For example, m6A methylation can control T-cell homeostasis (14). The antitumor effect of dendritic cells is regulated by mRNA m6a methylation and YTHDF1 (15). Furthermore, studies suggest that m6A methylation influences immunotherapy (16, 17). ALKBH5, a common m6A demethylase, was discovered to modulate the anti-PD-1 therapeutic response by inhibiting the accumulation of immune cells in TME in a study by Li et al (18). FTO may regulate melanoma response to immunotherapy (19). Nevertheless, Current studies on m6A methylation in glioblastoma are focus on individual m6A regulators and there is urgent need for an all-sided study of m6A-methylation modes in glioblastoma.

As a result, we used genomic data from six glioblastoma cohorts to evaluate m6A-methylation modes and correlate these patterns with TME infiltration traits in this study. We discovered two different m6A-methylation patterns and were surprised to discover that the TME features in these two patterns were very similar to immune tolerance and immunodeficiency phenotypes, respectively. Lasso analysis identified 14 prognosis-associated m6A methylation pattern signature genes. We generated a GM-score to evaluate the m6A-methylation patterns in individual patient using a principal component analysis (PCA) approach on the expression of the 14 m6A methylation pattern signature genes, demonstrating that m6A-methylation patterns contribute significantly in the characterization of the TME in glioblastoma. Briefly, our GM-score system enables more individualized and efficacious anti-tumor immunotherapy strategies by allowing us to identify TME infiltration features.

## Methods

### Data Collection and Processing

Publicly available gene expression data and complete clinical annotations were obtained from GEO and TCGA. Patients whose survival information were unavailable were excluded and 6 eligible GBM cohorts(TCGA-GBM, GSE7696, GSE13041, GSE72951, GSE83300, GSE122586) selected for further analysis. The “ComBat” algorithm using the sva package corrected for batch effects due to non-biotechnical bias. RNAseq transcriptome data for healthy human tissues were retrieved from GTEx. The GTEx and TCGA datasets were combined and then reconciled using quantile normalization as well as svaseq-based batch effect removal. CNV data were retrieved from Xena. As previously reported, m6A regulator mutation rates and CNV frequencies were calculated (20–22). For validation purposes, CGGA transcript data can be downloaded from the CGGA website.

### Immunohistochemistry

Immunohistochemistry results were obtained from the Human Protein Atlas (HPA) database (https://www.proteinatlas.org/). The Human Protein Atlas, one of the world’s most accessed biological databases, is a project to map the entire human proteome using proteomics and the integration of various other histological techniques. Immunohistochemical Average Optical Density values were measured with Image-Pro Plus6.

### Unsupervised Consensus Clustering Based on 15 m6A Regulators

According to expression levels of 15 m6A regulators, including 4 writers (METTL3, WTAP, ZC3H13, RBM15B), 1 eraser (FTO), and 10 readers (YTHDC1, HNRNPA2B1, YTHDC2, HNRNPC, YTHDF3, FMR1, IGFBP1, LRPPRC, IGFBP2, IGFBP3), unsupervised clustering analysis was used for identification of various m6A modification patterns and patient classification for further analyses. Cluster numbers and their stabilities were evaluated using a consistent clustering algorithm.

### Gene Set Variation Analysis

Gene set “c2.cp.kegg.v6.2.symbols” was retrieved from the MSigDB and GSVA enrichment analysis used to identify different m6A methylation patterns using “GSVA” package on R, with adjusted *p=*<0.05 indicating statistical significance. In non-parametric and unsupervised approaches, GSVA is used for estimation of pathway and biological process changes in gene expression datasets.

### Estimation of TME Immune Trait

The enrichment fraction from ssGSEA analysis was used to indicate relative abundances of every TME-infiltrating cell per sample. The genomes used to mark every TME-infiltrating immune cell type were retrieved from Charoentong’s study, which annotated human immune cell subtypes, such as activated CD8 T-cells, activated dendritic cells, macrophages, natural killer T-cells, and regulatory T-cells. Immune checkpoint, EMT, Pan-F-TBRS were also included.

### Construction of Glioblastoma m6A Scoring System

Based on 15 m6A regulators, patients were classified into 2 m6A methylation modification patterns. Identification of DEGs between the different patterns was done by Empirical Bayesian analysis using the limma package in R. Lasso analysis and univariate Cox regression analysis of DEGs were performed to identify prognosis-related m6A methylation pattern signature genes, which were then subjected to PCA to establish GM-score.


$$\text{GM}-\text{score}=\sum (\text{PC}1_{\text{i}}+\text{PC}2_{\text{i}})$$

### Statistical Analysis

The limma R package was used to perform differential gene expression analysis. The Spearman method was used for correlation analysis. To compute composition differences, Pearson’s chi-square analysis was used. The Wilcoxon rank sum test was used to calculate the statistical difference between the two groups. The Kruskal– Wallis test was used for comparisons of more than two groups. R software was used for all statistical analyses.

## Results

### Landscape of m6A Methylation Regulators in GBM

**Figure 1:**
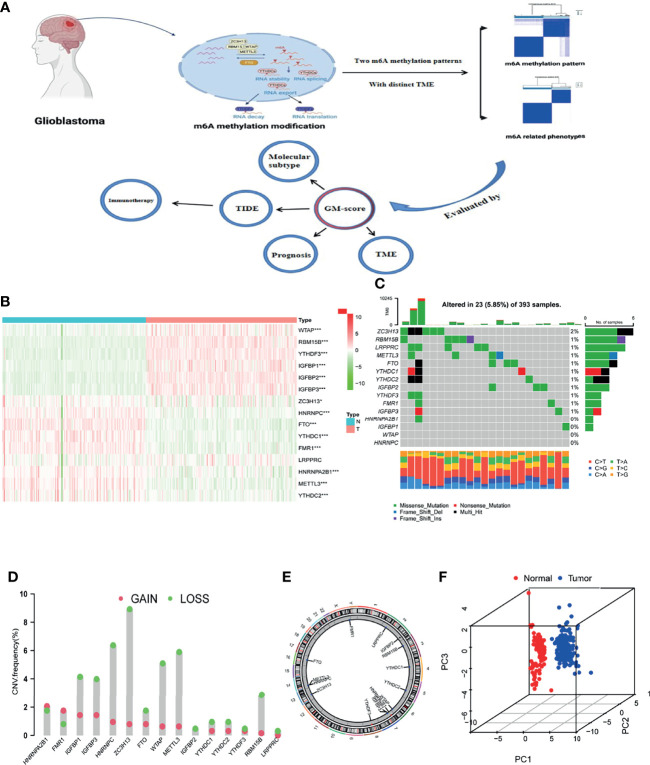
Landscape of m6A methylation regulators in GBM. **(A)** Graphical summary of this study. The graphical summary was created with BioRender. **(B)** Heatmap of differential expression of m6A methylation regulators (Normal= 160, Tumor = 169; Normal sample from GTEx dataset, Tumor sample from TCGA-GBM dataset). **(C)** Mutation frequency of 15 m6A methylation regulators. **(D)** CNV variation frequencies of m6A methylation regulators in the TCGA-GBM cohort. Column height represents change frequency. Green indicates loss. Red indicates gain. **(E)** Chromosomal locations of altered CNV in the m6A regulators in the TCGA-GBM cohort. **(F)** Principal component analysis of the 15 m6A methylation regulators in normal and GBM patients (Red indicates normal and blue indicates GBM patients; Normal sample from GTEx dataset, GBM sample from TCGA-GBM dataset). *, **, and *** indicate p = < 0.05, < 0.01, and < 0.001, respectively.

15 m6A methylation regulators (4 writers, 10 readers, and 1 eraser) were synthetically analyzed in this study. The graphical abstract of this research was depicted in **Figure 1A**. The overall design of this study is summarized in **Figure S1A**. Differential analysis of 15 m6A methylation regulators yielded that most regulatory factors were significantly differentially expressed in normal vs GBM tissues (*p*=<0.05, **Figure 1B**), and 11 regulators showed significant differences in the 4 subgroups (classical, mesenchymal, neural and proneural, **Figures S2A–O**). We then looked into the properties of the 15 m6A methylation regulators in greater detail. The 15 m6A regulators were altered 5.85 percent of the time in the 393 samples (23 mutations). The highest mutation rate was found in ZC3H13, followed by RBM15B (**Figure 1C**). Analysis of CNV alterations frequency revealed widespread CNV alterations (especially deletions) in the 15 regulators. While, HNRNPA2B1 and FMR1 had high CNV amplification frequencies (**Figure 1D**). The chromosomal locations of the CNV alteration on m6A regulators is shown on **Figure 1E**. PCA showed that expression levels of the 15 m6A methylation regulators could clearly distinguish between GBM and normal samples (**Figure 1F**). The results of the above analysis revealed that there was significant heterogeneity in the expression of m6A regulators between normal and GBM samples, implying that an imbalance in the expression of m6A regulators performs a vital part in the development of GBM.

### Protein Expression of m6A Regulators

Immunohistochemistry was used to confirm the differential expression of m6A regulators protein in normal brain tissue and glioblastoma. The expression of the remaining m6A regulators proteins, with the exception of HNRNPC and YTHDC1, was consistent with the above findings (**Figures S3A-J**).

### Two m6A Methylation Patterns of GBM

Six GBM cohorts were incorporated into a meta-cohort for further investigation of the role of the m6A methylation regulators in GBM. K-M analysis revealed that multiple m6A regulators had significant correlations with overall survival (**Figure S1B**). The comprehensive landscape of m6A regulator interactions, their prognostic significance and regulator connection in GBM patients was shown by an m6A regulator network (**Figure S1C**). The correlation of regulator co-expression was then investigated and significant correlation found between METTL3 and other regulators. The highest correlation (0.81) was between METTL3 and YTHDC2 (**Figure S1D**). Furthermore, we discovered that the writer and reader genes were differentially expressed in high and low FTO-expressing subgroups (**Figure S1E**). These results suggest that the expression of m6A regulators is significantly correlated, and thus we should investigate the role of m6A methylation modifications in glioblastoma from a comprehensive standpoint.

**Figure 2:**
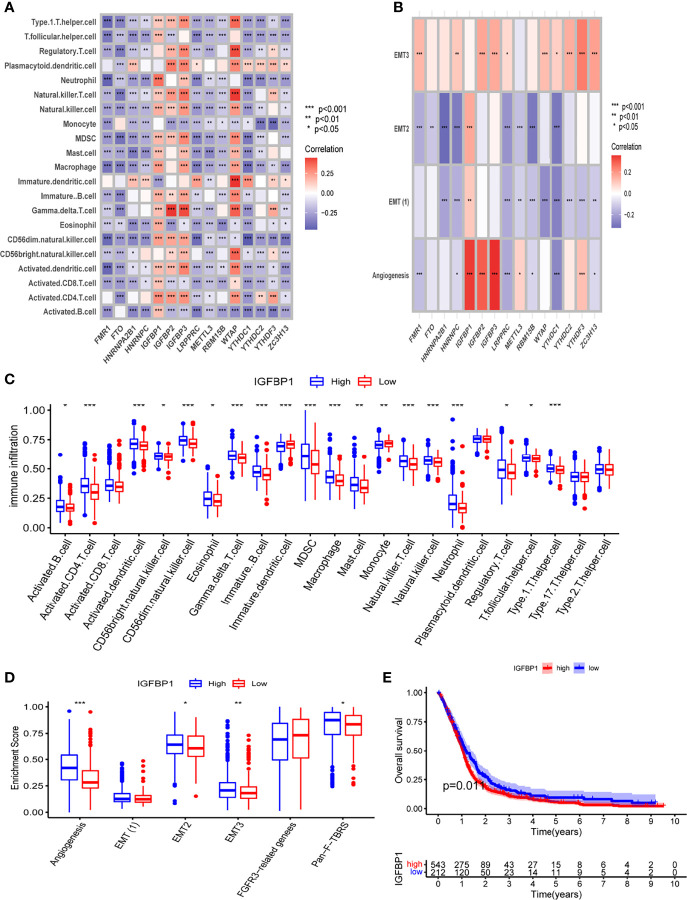
The relationship between TME and m6A regulators. **(A)** The correlation between m6A regulators and infiltrated immune cells of TME. **(B)** The correlation between m6A regulators and stromal of TME. **(C)** Differential analysis of immune cell infiltration in high and low IGFBP1 expression groups. **(D)** Differential analysis of stromal-related pathway in high and low IGFBP1 expression groups. **(E)** K-M analysis between high and low IGFBP1 expression groups. *, **, and *** indicate p = < 0.05, < 0.01, and < 0.001, respectively.

The specific relationship between TME and m6A regulators was then investigated. We observed that m6A regulators were significantly related to immune cell infiltration, with IGFBP1 was significantly positively related to immune cell and stroma-associated pathways (**Figures 2A, B**). We further compared the TME of patients with high and low IGFBP1 expression. Patients with high IGFBP1 expression had significantly higher immune cell infiltration and stroma-associated pathway activity than patients with low IGFBP1 expression (**Figures 2C, D**). Thus, in the m6A pattern, IGFBP1 may play a significant role in shaping the tumor microenvironment. Patients with low IGFBP1 expression also had a better prognosis (**Figure 2E**). The above findings imply that the m6A regulators may be important in the creation of distinct m6A modification patterns and immune characteristics of GBM.

**Figure 3:**
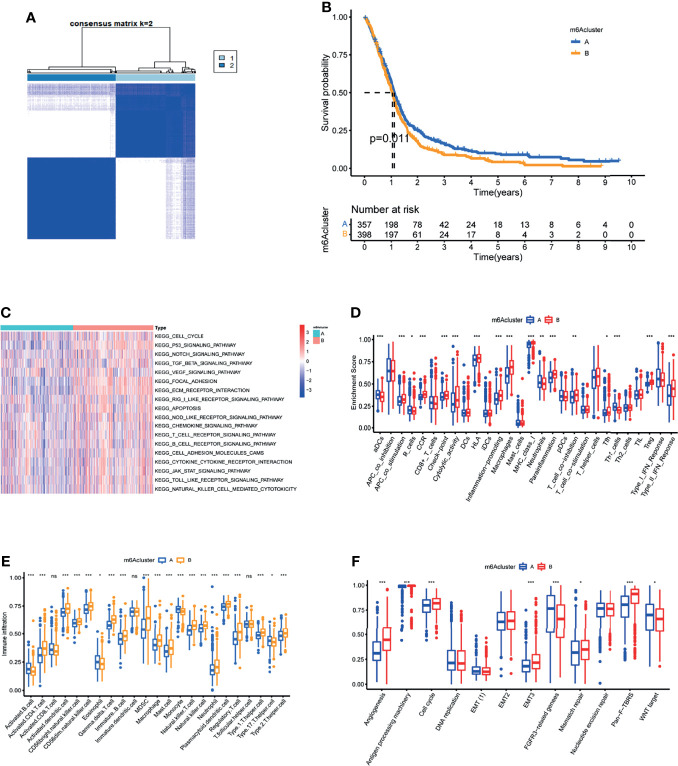
Characterization of the tumor microenvironment in the two m6A methylation patterns. **(A)** Unsupervised clustering analysis in GBM meta cohort. **(B)** Kaplan-Meier OS analysis in the two m6A clusters. P = 0.011. **(C)** GSVA enrichment analysis in the two m6A clusters. Red indicates pathway activation. Blue indicates pathway inhibition. **(D)** Differential of immune function between the two m6A clusters. **(E)** Abundance of Immune cell abundance in the two m6A clusters. **(F)**Enrichment score of common stromal activation pathways in the two m6A clusters.*, **, and *** indicate *p =* < 0.05, < 0.01, and < 0.001, respectively. ns, not significant. Upper and lower ends of the boxes indicate interquartile range. The lines in the boxes represent the median values.

Unsupervised consensus clustering of the expression of the m6A methylation regulators was used to investigate the m6A methylation patterns of glioblastoma. The consensus distributions for k (1-9) are displayed on empirical CDF plots (**Figure S4A**). Considering the consensus matrix for the analysis, k=2 was the best option. According to the consensus matrix, the unsupervised algorithm based on the 15 regulators clearly differentiated the samples, and each sample in a cluster had a strong association (**Figure 3A**). Therefore, based on expression levels of m6A methylation regulators, GBM patients were divided into 2 clusters (A and B). Further, we found that the prognoses for these patients markedly differed, with cluster A patients exhibiting better OS (**Figure 3B**).

### TME Features in the Two m6A Clusters

GSVA enrichment analysis of biological behavior differences between the 2 clusters showed that stromal as well as immune activation pathways like ECM receptor interactions, TGFβ signaling, cytokine-cytokine receptor interaction, cell adhesion chemokine signaling, T-cell receptor signaling, and Toll-like receptor signaling were significantly enriched in m6A cluster B (**Figure 3C**). While immune-related biological processes are lowly expressed in m6A cluster A (**Figure 3C**). Moreover, common immune functions like APC co stimulation, proinflammation, and type II IFN response are lower in m6A cluster A patients (**Figure 3D**). To elucidate the relationship between the m6A clusters and GBM immune traits, we analyzed infiltrating immune cells in every cluster. Surprisingly, m6A cluster B contains almost all adaptive and innate immune cells, with the exception of activated B cells, eosinophils, monocytes, and Th17 cells (**Figure 3E**). While patients in m6A cluster B did not show a corresponding survival advantage. Previous research has revealed that stromal cells in tumors play an immune-regulatory role (23). On the one hand, stromal cells can prevent immune cells from entering the tumor parenchyma. Stromal cells, on the other hand, can inhibit the function of T cells to kill tumor cells (21). Furthermore, tumor cells and stromal cell-driven angiogenesis have been shown to promote tumor metastasis (24). GSVA analysis results revealed significantly enhanced activity of stroma-related pathways in m6A cluster B (**Figure 3C**). As a result, we hypothesize that stromal activation in cluster B is the primary cause of immunosuppression and poor prognosis in glioblastoma. Subsequent analysis showed that stroma-related activities like angiogenesis, EMT, and Pan F TBRS were significantly enhanced in cluster B, which confirmed our speculation (**Figure 3F**). Taken together, we found that in GBM, the 2 m6A modification patterns have notably different TME cell infiltration characteristics. Cluster A was an immunodeficient phenotype exhibiting fewer immune cells and lower immune activity, while cluster B was an immune tolerance phenotype exhibiting increased immune cell infiltrations as well as stromal activation.

### Clinical Traits and Validation in the m6A Modification Patterns

The traitss of m6A-methylation patterns in distinct clinical features and biological behaviors were then investigated. Patients with the IDH1 mutant subtype mainly exhibited an immunodeficiency pattern in m6A cluster A, whereas patients with the IDH1 wild subtype were characterized by an immune tolerance pattern in m6A cluster B (**Figure S4B**). Furthermore, tumors with the m6A cluster A had better differentiation and were more likely to be proneural or neural in nature. While the m6A cluster B pattern had poorer tumor differentiation and was enriched in Mesenchymal and Classical subtypes (**Figure S4C**). IDH1 mutation molecular subtypes and Proneural histological types were significantly associated with better survival in glioblastoma, whereas IDH1 wild molecular subtypes and Mesenchymal histological types were significantly associated with worse clinical outcome (25, 26). Tumors with m6A cluster B had stromal activation, were highly malignant, and progressed quickly. These results again confirmed that m6A cluster B is significantly associated with stromal activation.

To validate the accuracy of the m6A methylation pattern classification, 373 glioblastoma patients from the CGGA database were chosen. Unsupervised clustering discovered two completely different m6A modification patterns in CGGA cohort, similar to meta cohort clustering (**Figure S4D**). Similarly, m6A cluster B in CGGA has increased immune infiltration and stromal activation, as well as a poor prognosis (**Figures S4E–G**). The above results verified the accuracy of m6A methylation pattern classification.

### Characteristics of m6A Related Phenotypes

**Figure 4:**
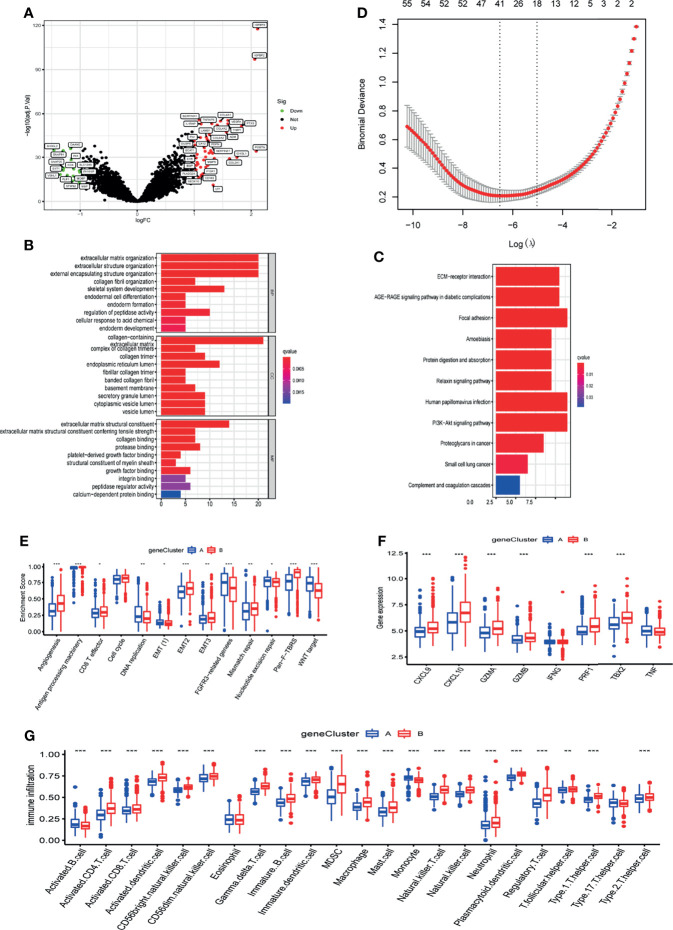
Characteristics of m6A related phenotypes. **(A)** DEGs associated with the m6A phenotype. **(B)** GO enrichment analysis of differential genes (DEGs). **(C)** KEGG enrichment analysis of differential genes (DEGs). **(D)** Lasso analysis was used to obtain 41 signature genes of m6A methylation pattern. **(E)** The expression of common signatures in 2 m6A gene-clusters. **(F)** The expression of common immune activation genes in the 2 gene-clusters. **(G)** Abundance of Immune cell abundance in the 2 gene-clusters. *, **, and *** indicate p = < 0.05, < 0.01, and < 0.001, respectively.

To further analyse the underlying biological processes of the two m6A modification patterns, we identified 58 DEGs associated with the m6A phenotype using the limma package (**Figure 4A**). GO and KEGG pathway enrichment analyses of the DEGs surprisingly showed that they were enriched in stromal activation pathways, confirming that m6A methylation modifications are closely linked to TME regulation (**Figures 4B, C**). To further validate the regulatory mechanism of m6A methylation modification in the tumor microenvironment of glioblastoma, we first performed Lasso analysis on 58 DEGs to obtain 41 signature genes of m6A methylation pattern, and then classified patients into different gene-clusters based on unsupervised clustering analysis of these genes (**Figure 4D** and **Table S1**). Consistent with cluster grouping of m6A methylation regulators, unsupervised clustering algorithm classified GBM patients into 2 classes based on these signature genes, which were named m6A gene-clusters A and B (**Figure S5A**). These results demonstrated that glioblastoma does have two different m6A methylation modification patterns.

Most m6A regulators were expressed differently in the two gene-clusters, which is in line with the m6A methylation pattern’s outcomes (**Figure S5B**). Similarly, the IDH1 mutation molecular subtype and the better differentiated Proneural tissue subtype were predominantly enriched in gene-cluster A, whereas the IDH1 wild molecular subtype and the poorly differentiated Mesenchymal tissue subtype were predominantly enriched in gene-cluster B (**Figures S5C, D**). gene-cluster A had a better prognosis than gene-cluster B,which is also in line with the m6A methylation pattern’s expected outcomes (**Figure S5E**).

To verify the relevance of m6A-related phenotypes to the tumor microenvironment, we analyzed the expression of common TME signatures in 2 m6A gene-clusters. Gene-cluster B had significantly higher stromal activity, as evidenced by the upregulation of angiogenesis and EMT signatures (**Figure 4E**). Meanwhile, immune activation-related signatures like Antigen processing machinery and CD8 effects also were found in abundance in gene-cluster B. These results suggest that the B gene-cluster Belongs to the immune tolerance phenotype. The expression of common immune activation genes and immune cell infiltration in the two gene-clusters was then compared (**Figures 4F, G**). The findings confirmed that gene-cluster B is immune tolerant phenotype and gene-cluster A is immunodeficient phenotype. These analyses again indicated that m6A methylation has a vital role in shaping the tumor microenvironment of GBM.

### Construction of Quantitative Model of m6A Methylation Modification Patterns in Individual Patients

**Figure 5:**
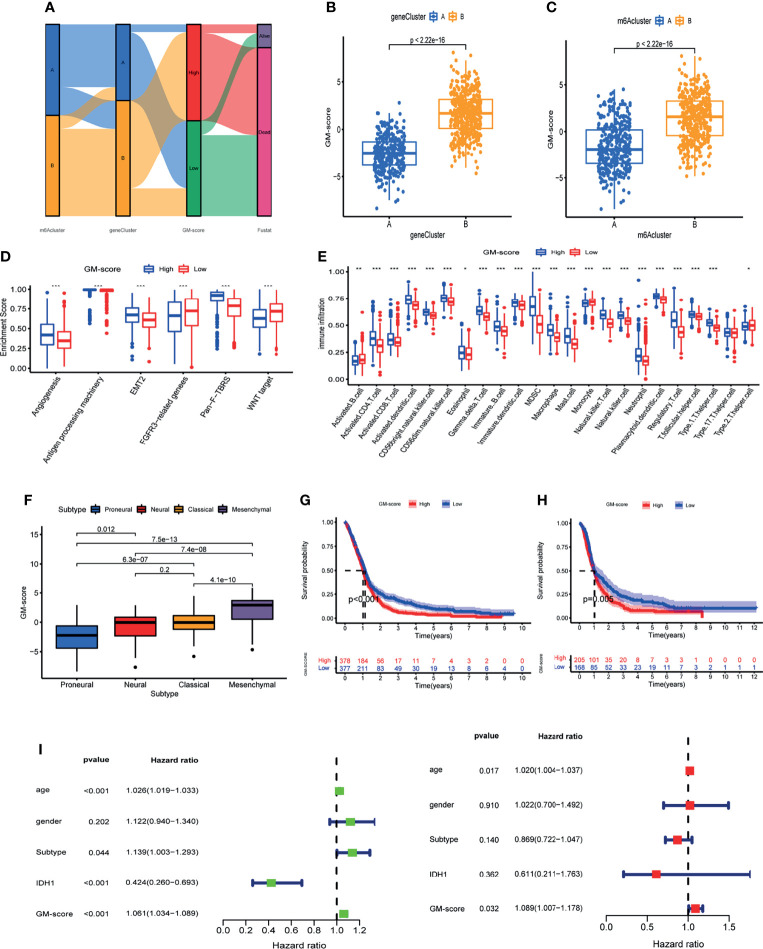
Construction of glioblastoma m6A scoring system (GM-score).**(A)** Alluvial diagram of GBM patient m6A cluster, m6A gene-cluster, and GM-score. **(B)** GM-score of the two m6A gene clusters. **(C)** GM-score of the two m6A clusters. **(D)** Degree of enrichment of stromal activation pathways in 2 GM-score subgroups. **(E)** Immune cell abundance in the 2 GM-score subgroups. **(F)** Differences in GM-score between various GBM subtypes. **(G)** Kaplan-Meier OS analysis of 2 GM-score subgroups in meta_cohort. **(H)** Kaplan-Meier OS analysis of 2 GM-score subgroups in CGGA cohort. **(I)** Independent prognostic analysis of GM-score. *, **, and *** indicate p = < 0.05, < 0.01, and < 0.001, respectively.

The analyses above were done on patient populations. Next, we sought to precisely quantify m6A methylation patterns in individual patients. Based on the univariate Cox analysis of 41 m6A methylation pattern signature genes, we obtained 14 prognosis-related signature genes (**Table S2**). Based on the 14 prognosis-related m6A methylation pattern signature genes, we created a glioblastoma m6A scoring system(GM-score) by PCA to assess m6A methylation modifications in individual patients. This scoring system takes full account of individual patient heterogeneity and can effectively assess patients’ m6A methylation patterns. Patients were assigned into high- and low-GM-score subgroups based on median score. The Alluvial diagram revealed the variation of m6A cluster, gene-cluster and GM-score in individual patients and also demonstrated the consistency and reliability of our analysis results (**Figure 5A**). The GM-score of gene-cluster B was higher than that of gene-cluster A, implying that the high score is linked to immune and stromal activation signals (**Figure 5B**). Importantly, the GM-score of m6A cluster B was also higher than that of m6A cluster A (**Figure 5C**). Further investigation revealed that high scores were indeed related to increased stromal activity and immune infiltration (**Figures 5D, E**). Furthermore, patients with the Mesenchymal subtype had a higher GM-score than patients with the other three tissue subtypes (**Figure 5F**). These observations imply that having a high-GM-score is associated with immune and stromal activation. GM-score can better assess the m6A methylation modification pattern in individual patients and further evaluate the TME characteristics to distinguish the nature of immunosuppression in individual patients.

Following that, we attempted to identify the utility of GM-score in predicting patients’ prognosis. Patients with low-GM-score had higher survival rates and longer survival periods than patients with high-GM-score, according to Kaplan-Meier OS curves (**Figure 5G**). We calculated the GM-score of GBM patients in the CGGA database and divided them into high and low subgroups based on the same median to test the model’s reliability and stability. Patients with a low-GM-score had a better prognosis, according to the findings (**Figure 5H**). We verified if GM-score could be used as a standalone prognostic biomarker for glioblastoma. A multivariate Cox analysis that included age, gender, IDH1 status, and histological type confirmed GM-score as a reliable and independent marker for determining prognosis (**Figure 5I**).

### GM-Score in the Role of Immunotherapy

**Figure 6:**
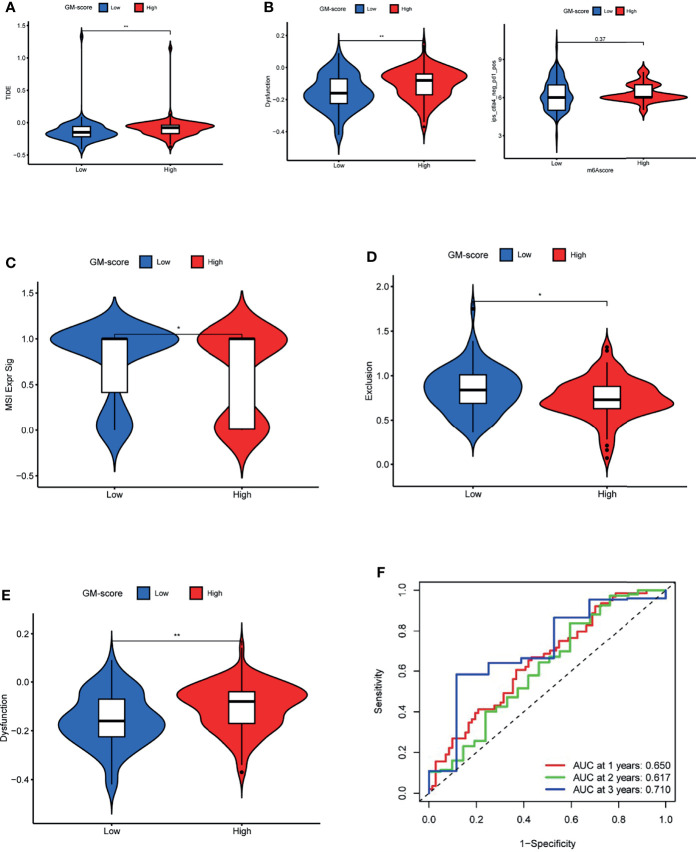
GM-score in the role of immunotherapy. **(A)** TIDE score of the 2 GM-score subgroups. **(B)** Effectiveness of immunotherapy in GM-score subgroups. **(C)** MSI score of the 2 GM-score subgroups. **(D)**T-cell exclusion score of the 2 GM-score subgroups. **(E)** T-cell dysfunction score of GM-score subgroups. **(F)** ROC curves of GM-score for predicting 1-, 2-, and 3-year survival. *, **, and *** indicate p = < 0.05, < 0.01, and < 0.001, respectively.

Because TCGA-GBM patients have complete immunotherapy information, we investigated the role of GM-score in predicting immunotherapy response in the TCGA cohort. TIDE was used to predict immunotherapy response in different GM-score subgroups. Patients with high TIDE scores had a lower response to immunotherapy, indicating that immunotherapy was less likely to benefit them (27). TIDE scores were lower in the low-GM-score subgroup than in the high-GM-score subgroup, implying that ICI treatment may benefit patients in the low-GM-score subgroup more (**Figure 6A**). To verify this conclusion, we compared the effect of anti-CTLA4 and anti-PD1 treatment on different GM-score subgroups (**Figure 6B**). The anti-CTLA4 treatment works better in patients with low-GM-scores, whereas anti-PD1 immunotherapy doesn’t show a similar result.

To determine the cause of the differences in immunotherapy response, we compared microsatellite instability (MSI), T-cell exclusion, and T-cell dysfunction scores between the two subgroups. The low-GM-score subgroup had higher scores for microsatellite instability (MSI) and T-cell exclusion, whereas the high-GM-score subgroup had higher scores for T-cell dysfunction (**Figures 6C–E**). In addition, ROC analysis revealed that the AUC values of GM-score reached 0.650 at 1 year, 0.617 at 2 years, and 0.710 at 3 years (**Figure 6F**).

## Discussion

As the most common type of epigenetic modification, m6A methylation modifications may play a vital role in shaping of the tumor microenvironment (28). Most studies, however, have focused on the role of single m6A regulators in tumors, and the role of m6A methylation modifications in tumor development has rarely been evaluated holistically. We systematically described TME cell infiltration mediated by the combined action of multiple m6A regulators, as well as the corresponding modification patterns, in this study. Actually, two distinct m6A modification patterns have been identified as important in shaping the TME landscape. Furthermore, a scoring system known as GM-score was developed to correlate individual patients’ m6A modification patterns with their immunotherapy response, offering a clinical instrument for a more personalized and efficient antitumor immunotherapeutic strategy.

Herein, we initially assessed the expression levels, somatic mutations, and copy number changes of 15 m6A regulators in glioblastoma patients and discovered that 14 m6A regulators had differential expression. Among all m6A regulators, ZC3H13 had the highest mutation frequency. Furthermore, the majority of the m6A regulators had copy number changes. These findings imply that an imbalance in the expression of m6A regulators may play an important role in the development of GBM. Huang et al. discovered that YTHDF2 promotes aggressive glioblastoma growth (29). Chen’s research suggests that m6A regulators contribute to malignant progression and have an effect on glioblastoma clinical prognosis (30). The correlation between m6A regulators and glioblastoma tumor microenvironment infiltrating cells, however, remains unknown. As a result, we investigated the correlation between the 15 regulators and glioblastoma tumor microenvironment. We discovered a strong relationship between these regulators and glioblastoma tumor microenvironment immune infiltrating cells, with IGFBP1 being significantly positively correlated with most immune cells and stroma-associated pathways, implying a potential role of m6A methylated modification in the formation of tumor microenvironment in glioblastoma.

Through unsupervised clustering analysis of expression of 15 m6A regulators, we developed 2 distinct m6A methylation modification patterns in glioblastoma and labeled them m6A clusters A and B. TME cell infiltration analysis, to our surprise, revealed that m6A cluster B was comparatively enriched in innate immune cell infiltration, such as monocytes, macrophages, and dendritic cells, and also adaptive immune cell infiltration. Patients with m6A cluster B, however, did not have a corresponding survival advantage. Previous research has demonstrated that stromal cells play an immunomodulatory role in tumors (31). On the one hand, stromal cells can keep immune cells out of the tumor parenchyma (21). Stromal cells, on the other hand, can prevent T cells from killing tumor cells (24). Furthermore, tumor cells and stromal cell-driven angiogenesis have been linked to tumor metastasis. As a result, we hypothesized that activation of stroma in cluster B inhibited immune cell anti-tumor effects. Several studies have found that inflammatory infiltration by tumor-associated macrophages promotes glioblastoma growth and metastasis, resulting in a poor prognosis (32). Importantly, the interaction of tumor-associated macrophages with stromal cells contributes to the formation of the tumor microenvironment (33). As a result, therapies that target tumor-associated macrophages may improve patient prognosis (34). Cluster A had less immune cell infiltration, including both intrinsic and adaptive immune cells, than cluster B. Not surprisingly, survival in cluster B was lower due to suppression of innate and adaptive immunity, but it was higher in comparison to cluster A. The GSVA analysis revealed that the two m6A modification patterns followed significantly different pathways. ECM receptor interaction, TGF signaling, cytokine-cytokine receptor interaction, cell adhesion chemokine signaling, t-cell receptor signaling, and toll-like receptor signaling were all significantly enriched in m6A cluster B. Moreover, we discovered that IDH1 mutant molecular subtype and proneural tissue subtype dominated m6A cluster A, whereas IDH1 wild subtype mesenchymal tissue subtype dominated m6A cluster B. We repeated the analysis on the CGGA cohort to confirm the accuracy of m6A methylation pattern classification and obtained the same results.

Following that, a Lasso analysis of DEGs between m6A methylation modification patterns identified 41 m6A pattern signature genes that may be mediated as post-transcriptional modification products by m6A regulators. Similar to the m6A modification pattern clustering results, two gene-clusters were associated with different TME-infiltrating cells, tumor subtypes, and prognosis. The complexity of m6A modification patterns in individual patients necessitates a novel approach to quantifying m6A modification patterns. As a result, we developed the GM-score scoring system, which is based on 14 prognostically relevant m6A methylation pattern signature genes, to assess m6A methylation patterns in glioblastoma. Furthermore, the two GM-score subgroups have distinguishable TME infiltrative characteristics. In other words, the high-GM-score subgroup had more immune cells and higher stromal activity, whereas the low-GM-score subgroup had less immune cell infiltration. The low-GM score subgroup had a higher survival rate and a longer survival than the high-GM score subgroup, according to survival analysis. These findings suggest that the GM-score is a reliable and effective tool for clinical assessment of m6A modification patterns in individual patients, and that it can be used to assess the trait of TME-infiltrating cells in patients to indicate immunotherapy response.

Immunotherapy is effective in glioblastoma, but its efficacy has been inconsistent due to a lack of systematic knowledge of the immune milieu in GBM and the inability to assess individual patients’ immune status. TIDE was used to assess immunotherapy responsiveness in different GM-score subgroups. Patients in the high-GM-score subgroup had higher TIDE and T-cell dysfunction scores than patients in the low-GM-score subgroup, implying that their lower immunotherapy response could be due to immune evasion caused by T-cell dysfunction. The low-GM-score subgroup had higher MSI and lower TIDE scores than the high-GM-score subgroup, indicating less immune evasion and higher MSI. MSI-induced high mutational load has been shown to make tumors immunogenic and susceptible to immune checkpoint inhibitors (35–37). We directly compared the effect of anti-CTLA4 and anti-PD1 treatment on different GM-score subgroups. The anti-CTLA4 treatment works better in patients with low-GM-scores, whereas anti-PD1 immunotherapy doesn’t show a similar result. A study by Spencer et al. showed that anti-PD-1 primarily promotes the expansion of exhausted-like CD8 T-cell subpopulations in tumor infiltrates, whereas anti-CTLA-4 promotes the expansion of ICOS+ Th1-like CD4 T cells on top of that (38). We believe this may explain why anti-CTLA4 treatment works better in patients with low GM scores, whereas PD1/PD-L1 immunotherapy does not. Our research serve as the basis and provide a blueprint for better understanding patients’ antitumor immune responses, which can then be used to guide more personalized and efficient immunotherapy strategies.

Briefly, GM-score can systematically assess individual patients’ m6A methylation modification pattern and corresponding tumor microenvironment features in order to further determine the tumor’s immunophenotype and guide more effective clinical practice. Patients’ clinicopathological characteristics, such as histological subtypes, molecular subtypes, genetic variants, and MSI status, can also be assessed using GM-score. GM-score can also be used as a stand-alone prognostic biomarker to anticipate survival outcomes. The GM-score can also be used to predict patient response to anti-CTLA4 immunotherapy. More significantly, this research provided new insights into cancer immunotherapy by altering m6A modification patterns by targeting m6A regulators or m6A methylation pattern signature genes, reversing unfavorable cellular infiltration characteristics and converting cold tumors to hot tumors, which could aid in the development of new drug combination strategies or immunotherapeutic agents. Our research suggests new ways to improve patients’ clinical responses to immunotherapy, identify distinct tumor immune phenotypes, and promote tumor-specific immunotherapy.

## Conclusion

This study demonstrated that m6A modification patterns play an important role in the shaping of TME complexity and diversity. The GM-score could identify m6A modification patterns in individual patients, resulting in a more personalization and efficacious anti-tumor immunotherapy strategy.

## Data Availability Statement

The original contributions presented in the study are included in the article/**Supplementary Material**. Further inquiries can be directed to the corresponding author.

## Author Contributions

This piece was created by JF, WX, CL and BW combined and analyzed the data. This manuscript was written by ZZ, YZ and SW. All authors contributed to the article and approved the submitted version.

## Funding

This study was funded by the Natural Science Foundation of China (81772352), Changjiang Scholars Program (KY216R202103), Postgraduate Research & Practice Innovation Program of Jiangsu Province (KYCX21_1613, KYCX20_1434).

## Conflict of Interest

The authors declare that the research was conducted in the absence of any commercial or financial relationships that could be construed as a potential conflict of interest.

## Publisher’s Note

All claims expressed in this article are solely those of the authors and do not necessarily represent those of their affiliated organizations, or those of the publisher, the editors and the reviewers. Any product that may be evaluated in this article, or claim that may be made by its manufacturer, is not guaranteed or endorsed by the publisher.

## Supplementary Material

Supplementary Figure 1 Prognostic significance of 15 m6A regulators. **(A)** The overall design of this study. **(B)** Prognosis associated m6A regulators. **(C)** The comprehensive landscape of m6A regulator was shown by an m6A regulator network. **(D)** The correlation analysis of regulator co-expression.**(E)** Differential analysis of m6A regulators in high- and low- FTO expession subgroups.

Supplementary Figure 2 Landscape of 15 m6A regulators in TCGA subtypes of GBM.

Supplementary Figure 3 Immunohistochemistry of m6A regulators protein in normal brain tissue and glioblastoma.

Supplementary Figure 4 The m6A methylation patterns in CGGA cohort. **(A)** The consensus distributions for k (1-9). **(B)** Distribution of IDH1 molecular subtype in different m6A clusters. **(C)** Distribution of histological subtype in different m6A clusters. **(D)** Unsupervised clustering analysis in CGGA cohort. **(E)** Kaplan-Meier OS analysis in the two m6A clusters of CGGA cohort. **(F)** Immune cell abundance in the 2 m6A clusters of CGGA cohort. **(G)** Enrichment score of TME signatures in the 2 m6A clusters of CGGA cohort.

Supplementary Figure 5 Characteristics of m6A related phenotypes. **(A)** Unsupervised clustering analysis of 41 signature genes of m6A methylation pattern.**(B)** Differential expression of 15 m6A regulators between the 2 gene-clusters.**(C)** Distribution of IDH1 molecular subtype in different gene-clusters.**(D)** Distribution of histological subtype in different gene-clusters. **(E)** Kaplan-Meier OS analysis in the gene-clusters.

Supplementary Table 1 The m6A methylation pattern signature genes.

Supplementary Table 2 The prognosis-related signature genes.
