# Supplementary figures and images for: N6-Methyladenosine Regulator-Mediated Immue Patterns and Tumor Microenvironment Infiltration Characterization in Glioblastoma
Source: Front Immunol. 2022 Mar 11;13:819080. doi: 10.3389/fimmu.2022.819080 (PMC8961865; doi:10.3389/fimmu.2022.819080)

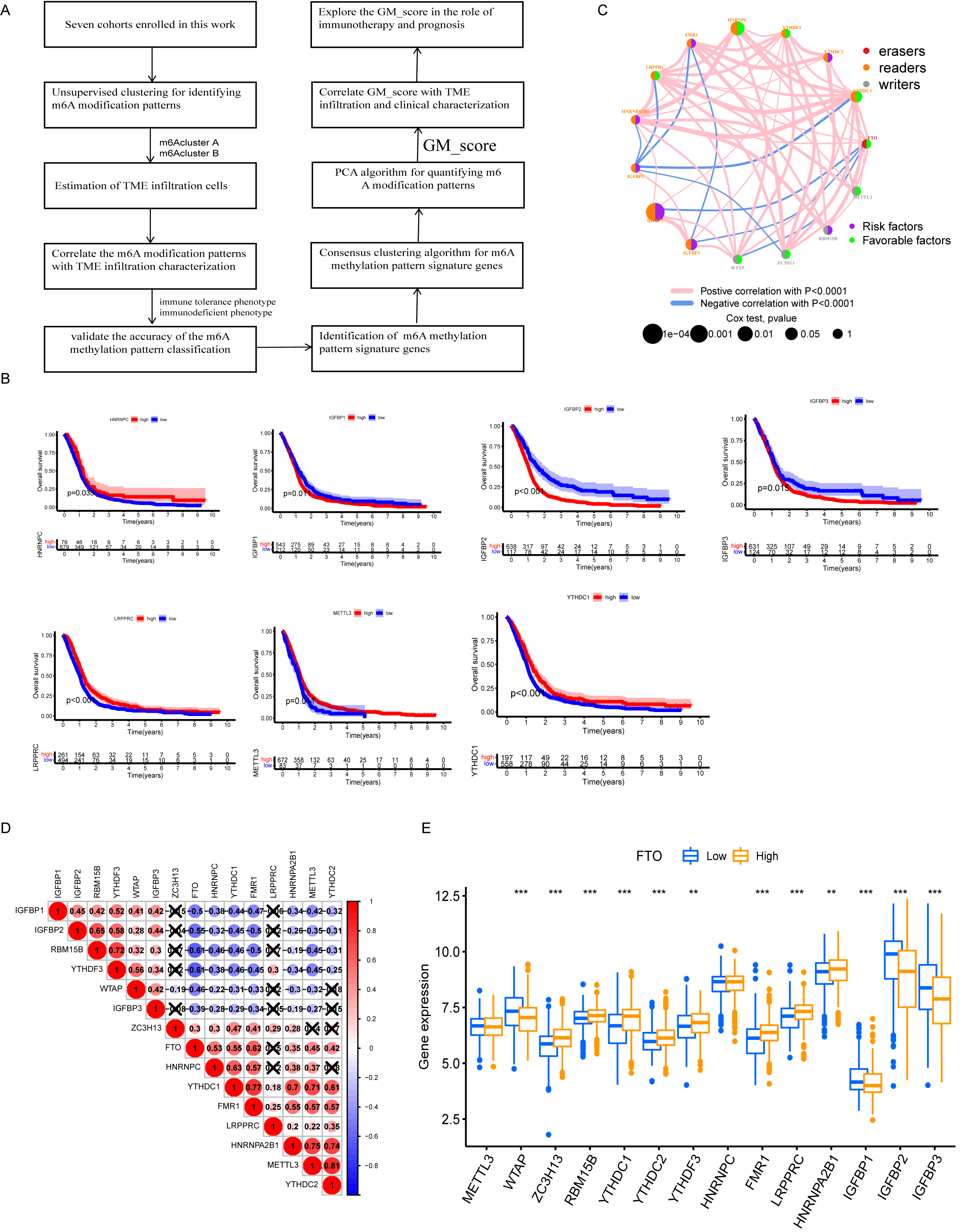

Supplement: Supplementary Figure 1 — Prognostic significance of 15 m6A regulators. (A) The overall design of this study. (B) Prognosis associated m6A regulators. (C) The comprehensive landscape of m6A regulator was shown by an m6A regulator network. (D) The correlation analysis of regulator co-expression.(E) Differential analysis of m6A regulators in high- and low- FTO expession subgroups. [file Image_1.tif]

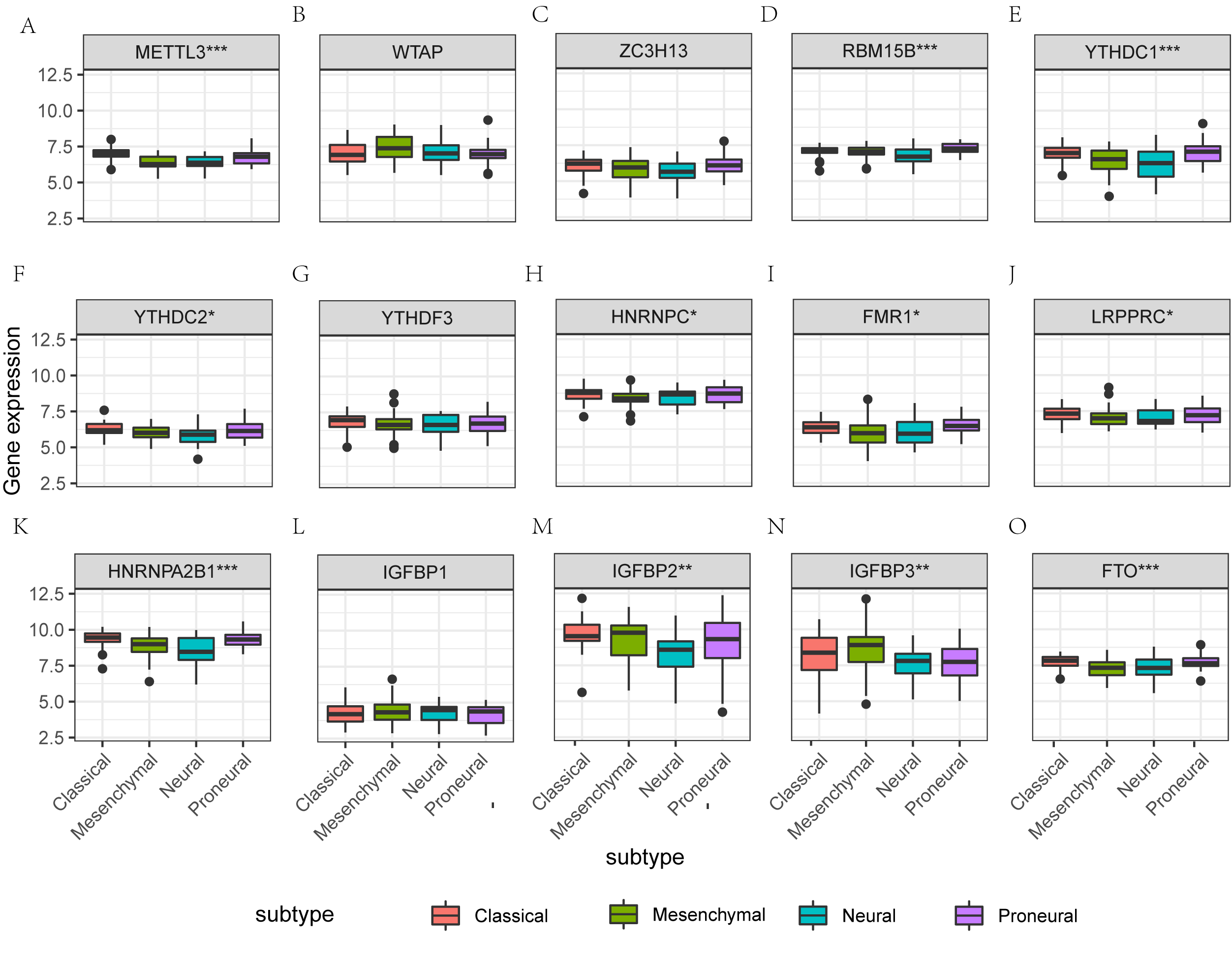

Supplement: Supplementary Figure 2 — Landscape of 15 m6A regulators in TCGA subtypes of GBM. [file Image_2.tif]

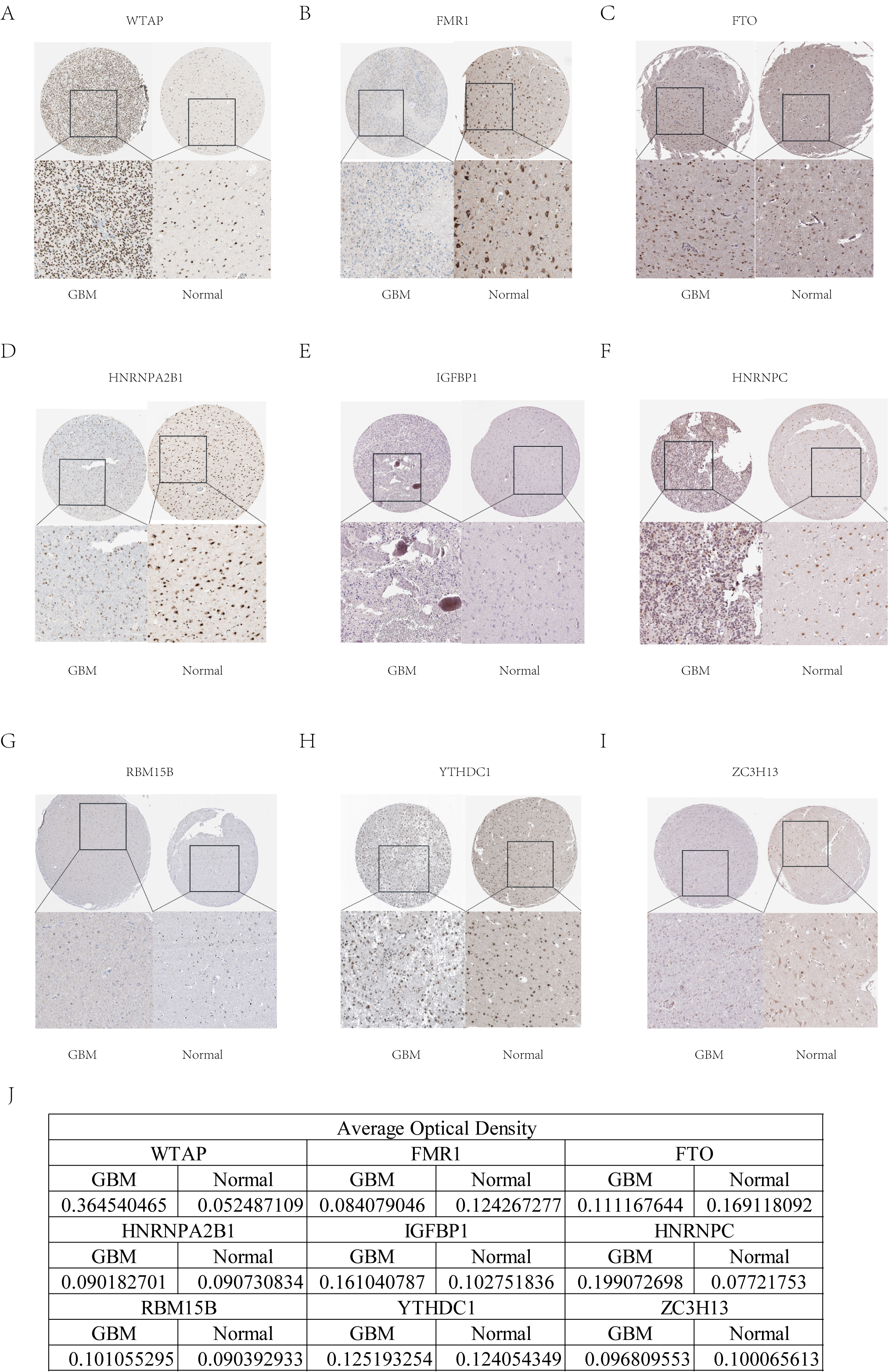

Supplement: Supplementary Figure 3 — Immunohistochemistry of m6A regulators protein in normal brain tissue and glioblastoma. [file Image_3.tif]

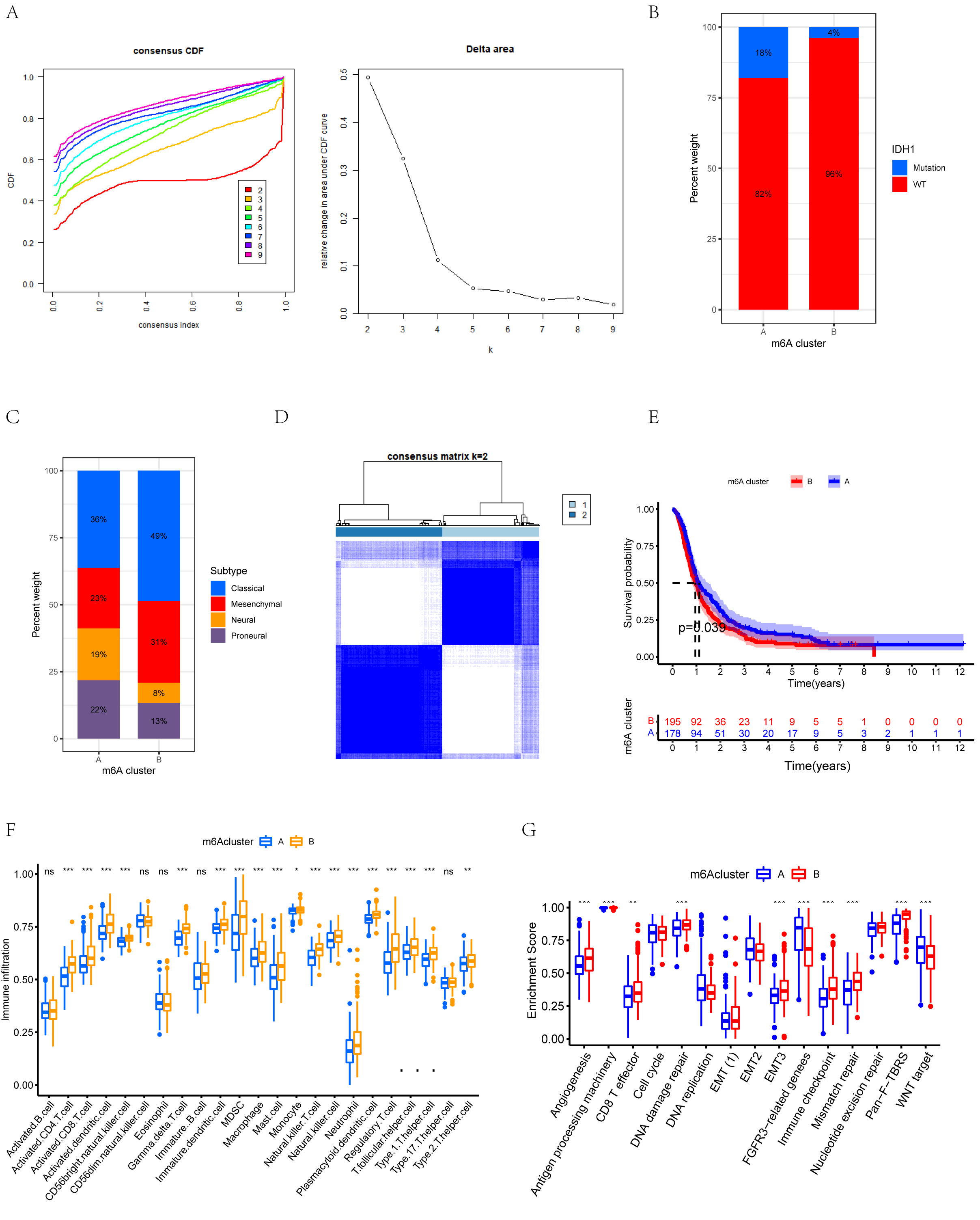

Supplement: Supplementary Figure 4 — The m6A methylation patterns in CGGA cohort. (A) The consensus distributions for k (1-9). (B) Distribution of IDH1 molecular subtype in different m6A clusters. (C) Distribution of histological subtype in different m6A clusters. (D) Unsupervised clustering analysis in CGGA cohort. (E) Kaplan-Meier OS analysis in the two m6A clusters of CGGA cohort. (F) Immune cell abundance in the 2 m6A clusters of CGGA cohort. (G) Enrichment score of TME signatures in the 2 m6A clusters of CGGA cohort. [file Image_4.tif]

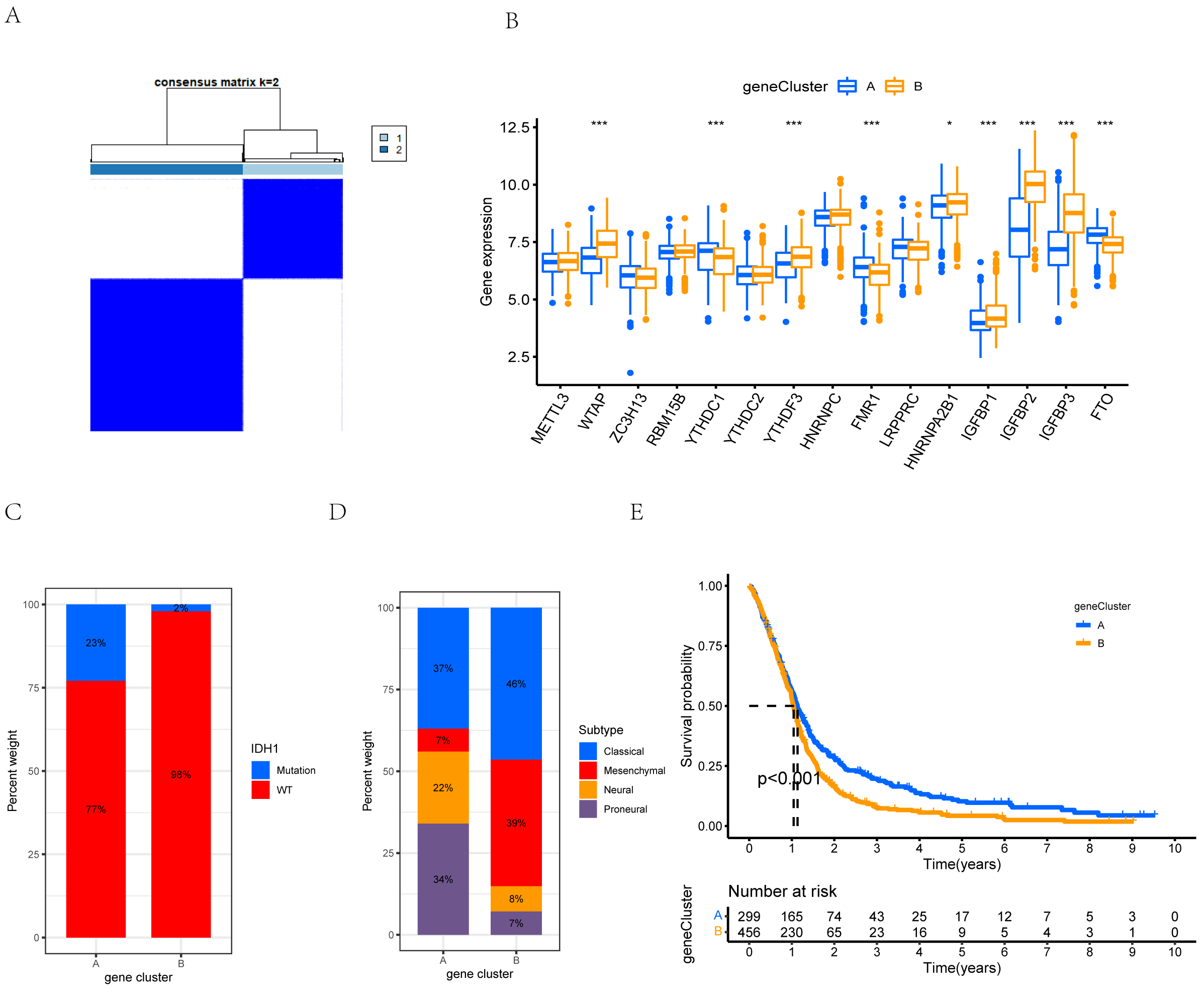

Supplement: Supplementary Figure 5 — Characteristics of m6A related phenotypes. (A) Unsupervised clustering analysis of 41 signature genes of m6A methylation pattern.(B) Differential expression of 15 m6A regulators between the 2 gene-clusters.(C) Distribution of IDH1 molecular subtype in different gene-clusters.(D) Distribution of histological subtype in different gene-clusters. (E) Kaplan-Meier OS analysis in the gene-clusters. [file Image_5.tif]
